# Stigma in health facilities: why it matters and how we can change it

**DOI:** 10.1186/s12916-019-1256-2

**Published:** 2019-02-15

**Authors:** Laura Nyblade, Melissa A. Stockton, Kayla Giger, Virginia Bond, Maria L. Ekstrand, Roger Mc Lean, Ellen M. H. Mitchell, La Ron E. Nelson, Jaime C. Sapag, Taweesap Siraprapasiri, Janet Turan, Edwin Wouters

**Affiliations:** 1 RTI International, 701 13th ST NW, Suite 750, Washington, DC, USA; 2 Epidemiology Department, UNC Gillings School of Global Public Health, 2103 McGavran-Greenberg Hall, CB #7435, Chapel Hill, NC 27599 USA; 3 Department of Global Health and Development, Faculty of Public Health and Policy, London School of Hygiene and Tropical Medicine, London, UK; 4 School of Medicine, Zambart, P.O. Box 50697, Lusaka, Zambia; 5 Division of Prevention Science, University of California, San Francisco, 550 16th Street, 3rd Floor, San Francisco, CA 94158-2549 USA; 6 St John’s Research Institute, St John’s National Academy of Health Sciences, Bengaluru, India; 7 Health Economics Unit, Centre for Health Economics, Faculty of Social Sciences, University of the West Indies, St. Augustine Campus, St. Augustine, Trinidad and Tobago; 8 International Institute for Social Studies, Erasmus University, Kortenaerkade 12, 2518 AX The Hague, Netherlands; 9 University of Rochester School of Nursing, 601 Elmwood Avenue, Box SON, Rochester, NY 14642 USA; 10 Centre for Urban Health Solutions, Li Ka Shing Knowledge Institute, St. Michael’s Hospital, 209 Victoria Street, Toronto, M5T 1B8 Canada; 11 Departments of Public Health and Family Medicine, School of Medicine, Faculty of Medicine, Pontificia Universidad Católica de Chile, Santiago, Chile; 12 Clinical Public Health Division, Dalla Lana School of Public Health, University of Toronto, Ontario, Canada; 13 Office of Transformative Global Health, Institute for Mental Health Policy Research, Centre for Addiction and Mental Health (CAMH), Ontario, Canada; 14 Department of Disease Control, Ministry of Public Health of the Government of Thailand, Tivanond Road, Nonthaburi, 11000 Thailand; 15 Department of Health Care Organization and Policy, Maternal and Child Health Concentration, School of Public Health, University of Alabama at Birmingham, Birmingham, USA; 16 Behavioral and Community Sciences Core, UAB Center for AIDS Research (CFAR), Birmingham, USA; 17 Centre for Longitudinal & Life Course Studies, University of Antwerp, Sint-Jacobstraat 2, B-2000 Antwerp, Belgium; 18 Centre for Health Systems Research & Development, University of the Free State, PO Box 399, Bloemfontein, 9300 South Africa

**Keywords:** Stigma, Discrimination, Reduction, Intervention, Programs, Health facilities

## Abstract

Stigma in health facilities undermines diagnosis, treatment, and successful health outcomes. Addressing stigma is fundamental to delivering quality healthcare and achieving optimal health. This correspondence article seeks to assess how developments over the past 5 years have contributed to the state of programmatic knowledge—both approaches and methods—regarding interventions to reduce stigma in health facilities, and explores the potential to concurrently address multiple health condition stigmas. It is supported by findings from a systematic review of published articles indexed in PubMed, Psychinfo and Web of Science, and in the United States Agency for International Development’s Development Experience Clearinghouse, which was conducted in February 2018 and restricted to the past 5 years. Forty-two studies met inclusion criteria and provided insight on interventions to reduce HIV, mental illness, or substance abuse stigma. Multiple common approaches to address stigma in health facilities emerged, which were implemented in a variety of ways. The literature search identified key gaps including a dearth of stigma reduction interventions in health facilities that focus on tuberculosis, diabetes, leprosy, or cancer; target multiple cadres of staff or multiple ecological levels; leverage interactive technology; or address stigma experienced by health workers. Preliminary results from ongoing innovative responses to these gaps are also described.

The current evidence base of stigma reduction in health facilities provides a solid foundation to develop and implement interventions. However, gaps exist and merit further work. Future investment in health facility stigma reduction should prioritize the involvement of clients living with the stigmatized condition or behavior and health workers living with stigmatized conditions and should address both individual and structural level stigma.

## Background

### Stigma defined

Stigma is a powerful social process that is characterized by labeling, stereotyping, and separation, leading to status loss and discrimination, all occurring in the context of power [1]. Discrimination, as defined by the Joint United Nations Programme on HIV/AIDS (UNAIDS), is the unfair and unjust action towards an individual or group on the basis of real or perceived status or attributes, a medical condition (e.g., HIV), socioeconomic status, gender, race, sexual identity, or age [2]. It has also been described as the endpoint of the stigmatization process [1]. Stigma is brought to bear on individuals or groups both for health (e.g., disease-specific) and non-health (e.g., poverty, gender identity, sexual orientation, migrant status) differences, whether real or perceived.

Health condition-related stigma is stigma related to living with a specific disease or health condition. Such stigma may be experienced in all spheres of life; however, stigma in health facilities is particularly egregious, negatively affecting people seeking health services at a time when they are at their most vulnerable. In health facilities, the manifestations of stigma are widely documented, ranging from outright denial of care, provision of sub-standard care, physical and verbal abuse, to more subtle forms, such as making certain people wait longer or passing their care off to junior colleagues [3–6]. As a result, stigma is a barrier to care for people seeking services for disease prevention, treatment of acute or chronic conditions, or support to maintain a healthy quality of life [7–19]. Within the health system, stigma towards a person living with a specific disease undermines access to diagnosis, treatment, and successful health outcomes [8, 20–28]. Stigma also impacts the well-being of the health workforce because healthcare workers may also be living with stigmatized conditions. They may conceal their own health status from colleagues and be reluctant to access and engage in care [4, 29–31]. Yet, stigma reduction is not a routine part of the way in which health services are delivered or evaluated, nor is it regularly integrated into pre-service and in-service training of all cadres of healthcare workers. This correspondence article explores how stigma is currently being addressed in health facilities across medical conditions, discusses gaps arising from a scan of the literature, and the potential for synergies across disease stigmas that could be harnessed for a joint response to more than one disease stigma. Specifically, for a variety of health conditions, we aimed to examine the health condition stigma addressed; intervention target populations, delivery, approaches, and methods; stigma drivers targeted; and evaluation methods and quality.

While recognizing that stigma is context-dependent, health condition stigmas in health facilities also display common features across countries and conditions in terms of certain stigma drivers, manifestations, and consequences [32–38]. This is particularly the case with stigma drivers, or factors considered to produce or cause stigma [3]. Within health facilities, common drivers can include negative attitudes, fear, beliefs, lack of awareness about both the condition itself and stigma, inability to clinically manage the condition, and institutionalized procedures or practices [3, 32, 35, 39–43]. Healthcare workers may fear infection, the behaviors of the stigmatized group (such as drug use or erratic or unpredictable actions), or mortality associated with the condition [3, 20, 32, 33, 35, 39, 40]. They may also experience moral distress based on their personal disapproval of behaviors associated with diseases, which may lead to stigmatizing reactions that impair their abilities to be effective providers, undermining quality of care [3, 20]. Healthcare workers may be unaware of how stigma manifests and affects people, and may therefore not be cognizant of the stigmatizing effects of their actions, or of how the health facilities’ policies or structures affect clients [3, 44, 45]. Lack of knowledge regarding the condition may also drive stigma [3, 38, 46]. For example, transmission misconceptions may drive stigmatizing, unnecessary precautions (e.g., double gloving, unnecessary quarantine), while disbelief in the curability of some stigmatized conditions may bias the provision of care [32, 35, 39]. Lacking knowledge about how to provide care for a specific condition, or lacking confidence in one’s ability to do so, may result in poor quality or discriminatory care [4, 20]. Institutional policies or systems for delivering care, such as verticalization (e.g., providing care at a separate clinic or “flagging” charts to distinguish them from the medical records of other patients) can also drive health facility stigma [3, 35].

The similarities are not only limited to drivers. The potential for generic survey tools to measure stigma (not specific to a particular health condition) was found in a literature review on leprosy, mental illness (MI), epilepsy, disability, and HIV [32]. Other studies have also found striking similarities in the consequences of stigma across diseases and cultures [15, 37, 47–49]. In many cases, clients might experience more than one type of stigma simultaneously (e.g., HIV or tuberculosis-related stigma, or substance use stigma) [42, 50–53].

While many health conditions are subjected to stigma, the following seven were selected as the focus of this correspondence article because of their high degree of commonality in stigma drivers: HIV, tuberculosis (TB), MI, substance abuse, diabetes, leprosy, and cancer [3, 32, 35, 39–41]. Having a negative attitude, in particular the culpability for the condition, is a driver for all seven of these conditions, as is lack of awareness of stigma and its consequences; level of knowledge, myths, and misbeliefs; and institutional policies, procedures, and practices [3, 32, 35, 39–43]. Fear of infection is common to four of the seven (HIV, TB, cancer, leprosy), while fear of the individual or their behavior is common to HIV, cancer, MI, and substance abuse [3, 20, 32, 33, 35, 39, 40].

In addition, although the specificities of the drivers, manifestations, and consequences of the stigmatization of different conditions can be varied (e.g., exactly what is feared), the mechanisms underlying the path between drivers, stigmatization, and its consequences often display universal characteristics. Theoretically, Link and Phelan [1] defined stigma as the co-occurrence of five components: labeling, stereotyping, separation, status loss, and discrimination [1]. The seven selected health conditions, which are stigmatized across a variety of contexts, display very similar mechanisms driving their stigmatization. Although the specific combined characteristics of a condition might be unique, the pathways through which these drivers feed the stigmatization of the seven selected conditions are often similar—especially in the specific context of health facilities.

The underlying shared mechanisms of the stigmatization process, common stigma drivers, the potential for generic health condition-related stigma measurement tools, the co-prevalence of stigmatized conditions (e.g., TB/substance abuse/HIV), and the similarities in the consequences of stigma, regardless of condition, all point to the potential for interventions to simultaneously reduce stigma related to more than one health condition at a time in health facilities. This would strengthen delivery of equitable, quality healthcare, while attending to the specific and important contextual or disease-conditions nuances.

This potential merits investigation, particularly in resource-constrained settings, where finding synergies for stigma reduction across conditions could create economies of scale, offering savings of cost and time. However, clearly, interventions must pay attention to specific cultural and socioeconomic contexts and recognize that stigmas are not always experienced in the same way in all settings.

An improved understanding of how health condition stigma is currently addressed in health facilities is needed to identify gaps and areas for investment in stigma reduction, as well as to explore the possibility of concurrently addressing more than one health condition stigma with a joint intervention. Thus, this correspondence article takes an explicitly programmatic focus and aims to examine “how” health facility-based stigma reduction interventions are implemented across health condition stigmas.

## Methods

### Article identification and selection criteria

Following the Preferred Reporting Items for Systematic Reviews and Meta-Analyses (PRISMA) guidelines [54], we searched PubMed, Psychinfo and Web of Science databases in February 2018. Gray literature was obtained from the United States Agency for International Development’s (USAID) Development Experience Clearinghouse. Additionally, literature was identified through expert consultation and an ancestry citation search.

The inclusion criteria were a clear description of (a) the implementation of an intervention that aimed to reduce one of the seven health condition stigmas in healthcare settings, either by targeting the potential perpetrators of stigma (healthcare workers or healthcare facility policies) or by empowering clients to overcome stigma and discrimination and (b) the evaluation (qualitative, quantitative, process, or mixed methods) of said intervention. We strove to capture all intervention approaches and implementation methods, regardless of the target population (health workers or clients). The search was restricted to articles published in the past 5 years in English. Reviews were excluded, as were articles that only described intervention development.

### Screening and data abstraction

Article citations and abstracts were organized, uploaded, and reviewed using EndNote. MS and KG screen abstracts to determine whether they included relevant information. The full text was obtained if at least one reviewer deemed the abstract to be relevant. MS and KG reviewed the full-text articles, and these were included if both reviewers agreed. Discrepancies were discussed with LN until a consensus was reached. Finally, MS and KG conducted ancestry searches of the citations of included articles. Data were abstracted using a standardized abstraction form adapted from a systematic review of interventions to reduce HIV-related stigma by Stangl et al. [55]. Specifically, we aimed to examine the health condition stigma addressed; the intervention populations, delivery, approaches, and methods; stigma drivers targeted; and the evaluation methods and quality.

### Data synthesis and quality assessment

Articles were categorized by disease-specific stigma addressed, approaches employed, intervention delivery, and stigma drivers addressed (Table 1). “Approaches” were considered as overarching strategies towards stigma reduction, and “methods” as the specific activities that reduce stigma.

**Table 1:** Study and intervention characteristics, stigma drivers, evaluation methods, and quality assessment score

First author, publication year, country, health condition	Intervention population, sample size	Stigma reduction approaches, duration	Brief intervention description	Stigma drivers targeted	Evaluation methods, quality score, effect on stigma
Aggarwal [103], 2013, USA, MI	Students, 250	PL, C; 2 h	Panel presentation and discussion	Attitudes, knowledge of stigma	QE/NC, 14/27, decreased
Bamgbade [104], 2017, USA, MI	Students, 120	I, PL, C; 2.5 h over 2 days	Presentations, videos, discussion and active-learning exercises	Attitudes, fear, knowledge of condition, knowledge of stigma	QE/NC, 15/27, decreased
Bamgbade [105], 2016, USA, MI	Students, 120	I, PL, C; 2.5 h over 2 days	Presentations, videos, discussion and active-learning exercises	Attitudes, knowledge of condition, knowledge of stigma	QE/NC, 16/27, mixed
Batey [74], 2016, USA, HIV	HCPs and PLHIV, 38	I, SB, PL, C, E; 1.5 day	Workshop	HCPs: attitudes, fear, knowledge of stigmaPLHIV: coping	QE/NC and qualitative, 13/27, mixed
Beaulieu [106], 2017, Canada, MI	HCPs, 111	I, PL, C; 3 3.5-h sessions over 2 months	Training modules led by consumer	Unclear	RCT, 22/27, Mixed
Bingham [107], 2018, New Zealand, MI	Students, 45	SB, PL, C; 12 h over 3 weeks	Guided clinical practice and discussion focused on attitudes and beliefs	Attitudes, fear, ability to manage condition	QE/NC, 10/27, mixed
Clarke [71], 2015, UK, MI	HCPs, 100	I, SB; 2 days over 2 weeks	Workshop	Attitudes, knowledge of condition, ability to manage condition	RCT and qualitative, 18/27, mixed
Economou [108], 2017, Greece, MI	Students, 678	I, SB, C; 120 h over 4 weeks	Lectures and clinical placement	Knowledge of condition, ability to manage condition, unclear	QE/NC, 18/27, decreased
Feeney [65], 2013, Ireland, substance abuse	Students, 119	SB, PL, C; 6 weeks	Clinic posting, patient presentations, discussion, assignments	Knowledge of condition, ability to manage condition	RCT and qualitative, 20/27, decreased
Fernandez [66], 2016, Malaysia, MI	Students, 102	I, PL, C; 3 h	Lecture, video or face-to-face presentation, discussion	Fear, knowledge of condition, knowledge of stigma	RCT, 17/27, decreased
Flanagan [67], 2016, USA, MI, substance abuse	HCPs, 27	C, PL; 1 h	Multimedia in-person performance by people living with a mental disorder	Fear, knowledge of stigma	RCT, 20/27, decreased
Friedrich [109], 2013, England, MI	Students, 1452	I, SB, PL, C; n/a	Lecture, testimonials, discussion, role-play providing clinical care	Knowledge condition, knowledge of stigma, ability to manage condition	QE/C, 15/27, mixed
Geibel [75], 2016, Bangladesh, HIV	HCPs, 300	I, SB, PL; 3 days	Workshop with lectures, discussion, participatory activities, & role-play providing clinical care	Attitudes, fear, knowledge of condition, knowledge of stigma, ability to manage condition	QE/NC, 15/27, decreased
Gulati [110], 2014, India, MI	Students, 135	SB, C; 2 weeks	Clinic posting	Ability to manage condition	Post, with control; 16/27; mixed
Happell [68], 2014, Australia, MI	Students, 201	SB, C; 12 weeks	Lecture delivered by stigmatized individual	Ability to manage condition	QE/C, 14/27, decreased
Hawke [89], 2014, Canada, MI	HCPs, students, clients, 137	C, PL; 50 min	Video performance and discussion	Knowledge of the condition	QE/NC and qualitative, 15/27, decreased
Iheanacho [111], 2014, Nigeria, MI	Students, 82	I, SB, PL; 4 days	lLctures, discussions, role-play providing clinical care	Knowledge of the condition	QE/NC, 15/27, mixed
Itzhaki [112], 2017, Israel, MI	Students, 101	I, SB, PL, C; 70 h over academic semester	Lectures, contact w/people with mental health disorders, skill building exercised, video on coping	Fear, knowledge of condition, ability to manage condition	QE/NC, 14/27, decreased
Jarvie [90], 2013, Canada, MI	Students, 49	PL, C; 2.5 h	Comedy show and discussion	Unclear	QE/NC, 16/27, mixed
Jaworsky [91], 2016, Canada, MI	Students, 67	SB, C; 2 h	Observed provision of HIV testing with PLHIV and testimonies	Ability to manage condition	QE/NC and qualitative, 14/27, decreased
Knaak [58], 2013, Canada, MI	HPCs and students, 58	I, PL, C; 2 h	Pamphlet, video screening of a play, discussion	Knowledge of condition	QE/NC, 13/27, decreased
Knaak [69], 2015, Canada, MI	HCPs, 230	I, SB, PL, C; 1 day	Workshop with lectures, skills training and testimonials	Attitudes, knowledge of condition, ability to manage condition	QE/NC, 13/27, decreased
Li [61], 2015, China, HIV	HCPs, 1760	I, PL, S; 12 months	Participatory training of champions from each hospital and provided universal precaution materials	Attitudes, fear, knowledge of condition, knowledge of stigma, ability to manage condition	RCT, 21/27, decreased
Li [62], 2013, China, HIV	HCPs, 1760	I, PL, S; 12 months	Participatory training of champions from each hospital and provided universal precaution materials	Attitudes, fear, knowledge of condition, knowledge of stigma, ability to manage condition	RCT, 22/27, decreased
Li [63], 2013, China, HIV	HCPs, 1760	I, PL, S; 12 months	Participatory training of champions from each hospital and provision of universal precaution materials	Attitudes, fear, knowledge of condition, knowledge of stigma, ability to manage condition	RCT, 23/27, decreased
Li [60], 2013, China, HIV	HCPs, 1760	I, PL, S; 12 months	Participatory training of champions from each hospital and provided universal precaution materials	Attitudes, fear, knowledge of condition, knowledge of stigma, ability to manage condition	RCT, 24/27, decreased
Li [63], 2013, China, HIV	HCPs, 1760	I, PL, S; 12 months	Participatory training of champions from each hospital and provided universal precaution materials	Attitudes, fear, knowledge of condition, knowledge of stigma, ability to manage condition	RCT, 24/27, decreased
Li [61], 2015, China, MI	HCPs, 77	I, SB, C; 85 h	Lectures, clinical placement,	Knowledge of condition, knowledge of stigma, ability to manage condition	QE/C, 18/27, decreased
Li [92], 2014, China, MI	HCPs, 99	I, PL, C; 1 day	Discussion and activities	Knowledge of condition, knowledge of stigma	QE/NC, 14/27, decreased
Lohiniva [93], 2016, Egypt, MI	HCPs, 347	I, SB, PL, C; 25 h over 4 months	Lectures, discussions, activities, training on universal precautions	Knowledge of condition, fear, knowledge of stigma	QE/C, 15/27, decreased
Lyons [113], 2015, Australia, MI	Students, 151 baseline, 161 follow-up	I, SB, C; 8 weeks	Lectures and clinical clerkship	Knowledge of condition, ability to manage condition	QE/NC, 15/27, decreased
MacCarthy [114], 2013, Canada, MI	HCPs, n/a	I, SB, PL; 1 day	Live or video lectures, discussion, and role-play service provision	Knowledge of condition, ability to manage condition	QE/NC, 7/27, decreased
Mak [72], 2015, Hong Kong, HIV	Students, 88	I, PL, or C; 1.5 h	Lecture and interactive game or in-person sharing session lead by PLHIV	Attitudes, knowledge of condition, knowledge of stigma	QE/NC, 17/27, decreased
Marzan-Rodriquez [115], 2016, Puerto Rico, HIV	Students, 20	I, SB, PL; 9 h over 3 days	Lectures, discussion, activities	Attitudes, knowledge of condition, knowledge of stigma	Process and qualitative, n/a
Michaels [116], 2014, USA, MI	HCPs, 131	I, PL; 3 h	Discussion, activities, video performance	Knowledge of condition, knowledge of stigma	RCT, 16/27, decreased
Morawska [117], 2013, Australia, MI, substance abuse	HPCs, educators, clients, 458	I, SB; 2 days	Workshop	Knowledge of condition, ability to manage condition	QE/NC, 13/27, decreased
Moxam [118], 2016, Australia, MI	Students, 79	PL, C; 5 days	Immersive camp outside of clinical setting	Unclear	QE/C, 15/27, decreased
Muzyk [119], 2017, USA, MI	Students, 74	I, PL; 6 sessions over 2 weeks	Discussion-based lectures with small group activities	Attitudes, knowledge of condition, knowledge of stigma	QE/NC, 12/27, mixed
Ng [27], 2017, Malaysia, MI	HCPs, 206	I, C; 5 min	Video	Fear, knowledge of condition	QE/NC, 17/27, decreased
Odeny [60], 2013, Kenya, HIV	PLHIV, 295	S; 12 months	Integration of HIV care with primary health care services	Institutionalized procedures	Repeated cross-sectional surveys, 17/27, mixed
Papish [70], 2013, Canada, MI	Students, 90	I, SB, PL, C; 4 weeks	Lecture, discussion, observed clinical care provision, videos, presentations	Attitudes, knowledge of condition, ability to manage condition	RCT, 21/27, decreased
Pulerwitz [64], 2015, Vietnam, HIV	Health facility staff, HCPs, 795	I, SB, PL, C; 1.5–2 days	Discussion, participatory activities, universal precaution skills building, development of a code of practice	Attitudes, fear, knowledge of condition, knowledge of stigma	QE/NC, 20/27, decreased
Shah [76], 2014, India, HIV	Students, 99	I, PL, C; 2 h over 2 weeks	Lectures, discussion, testimony	Attitudes, fear, knowledge of condition, knowledge of stigma	QE/C and process, 17/27, decreased
Shen [120], 2014, China, MI	Students, 325	SB, C; 8 week	Clinical clerkship	Ability to manage condition	QE/C, 14/27, decreased
Uebel [59], 2013, South Africa, HIV	HCPs and PLHIV, n/a	S; n/a	Integration of HIV care into primary health care	Institutionalized procedures	Process and Qualitative, n/a
Wakeman [121], 2017, USA, substance abuse	HCPs, 149	I, S; 1 year	Addition of services to improve care for substance abuse and an educational curriculum for providers	Knowledge about condition, unclear	QE/NC, 15/27, mixed
Winkler [73], 2017, Czech Republic, MI	Students, 60	I, PL, C; leaflet: n/a; in-person: 45 min; video: 7 min	Brochure, seminar discussion, or video	Attitudes, knowledge about condition, knowledge about stigma	RCT, 22/27, mixed

*Abbreviations*: *C* contact, *E* empowerment, *HCPs* health care providers, *HIV* human immunodeficiency virus, *I* information-based, *MI* mental illness, *PL* participatory learning, *PLHIV* people living with HIV, *QE/C* quasi-experimental with a control group, *QE/NC* quasi-experimental with no control group, *RCT* randomized controlled trial, *S* structural, *SB* skills building; students, students receiving healthcare training

MS and KG assessed the quality of quantitative data using the 27-item Downs and Black checklist [56]. Articles scoring 14 or above were considered high-quality studies [55]. The 18-item framework for evaluating qualitative evidence devised by Spencer et al. was used to assess the quality of qualitative data [57]. Studies scoring of 10 or above were considered high-quality studies [55].

## Results

### Stigma reduction in health facilities

**Fig. 1:**
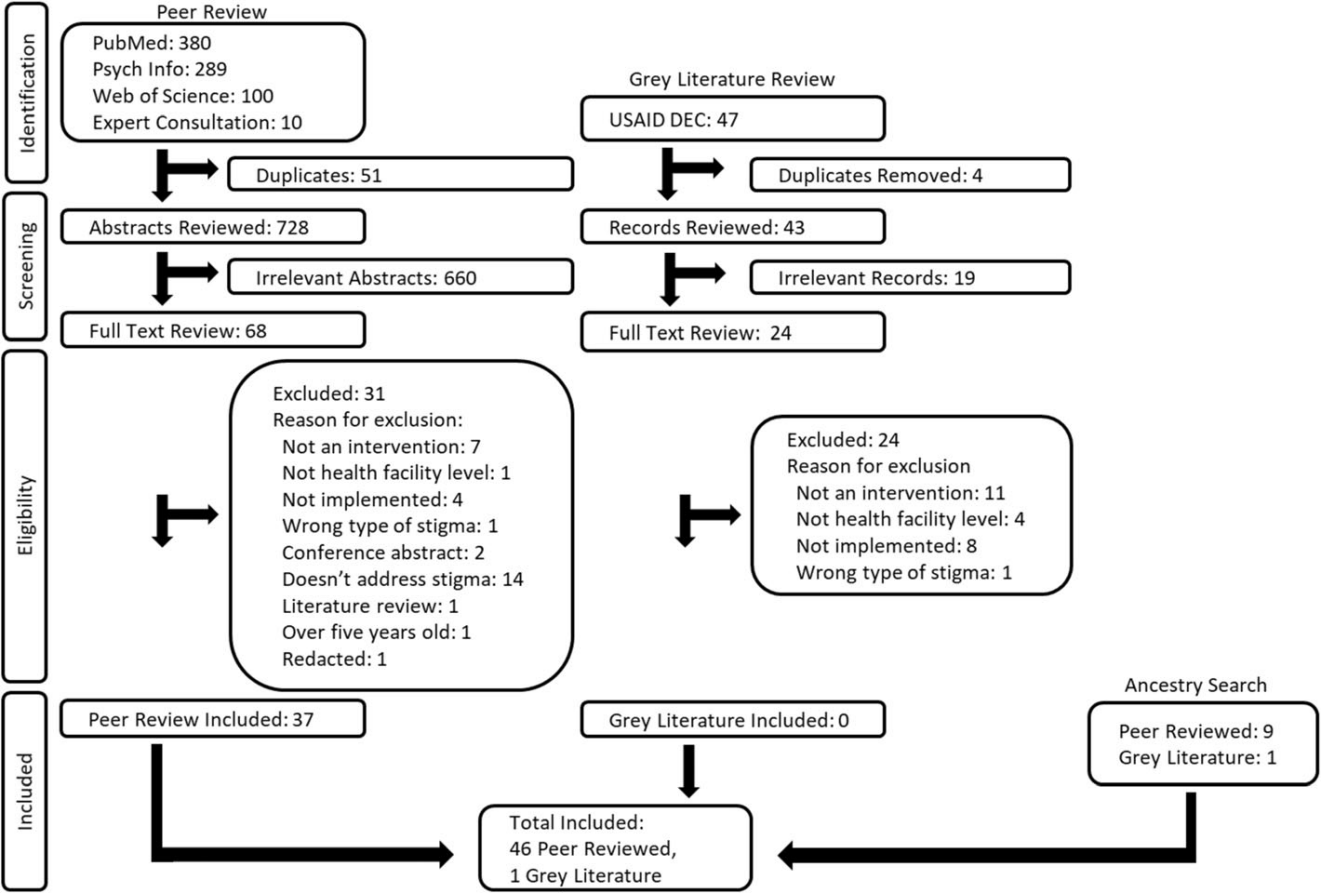
PRISMA flow diagram

A total of 728 peer-reviewed abstracts were assessed, of which 68 articles underwent full-text review and 37 met the inclusion criteria. All nine peer-reviewed records identified through a citation ancestry search were included. Forty-three gray literature records were reviewed, of which 24 underwent full-text review but none met the inclusion criteria. However, a project report identified through the ancestry search was included [58]. Forty-seven manuscripts detailing 42 distinct interventions were included (Fig. 1).

All the included interventions focused on stigma related to HIV, MI, or substance abuse. No articles meeting the inclusion criteria were found for TB, diabetes, cancer, or leprosy. Interventions that addressed more than one medical condition were only found for MI or substance abuse. Twenty of the identified interventions targeted healthcare providers, 24 targeted healthcare students, four included clients in the intervention population, and only one included all levels (medical and non-medical) of healthcare workers.

Most quantitative studies (38) scored at least 14 out of 27 points on the Black and Downs checklist and were thus categorized as high-quality studies for the purposes of this review. The scores ranged between 7 and 24, with an average score of 16.5. Over half of the interventions scored between 14 and 18 (*n* = 26). The two qualitative studies were categorized as being high quality (see Table 1 for individual study scores).

Interventions were implemented across the globe, with at least one intervention implemented in every World Health Organization region. The largest number (*n* = 16) were implemented in the Americas, eight in the USA (including one in Puerto Rico), and eight in Canada. Only one intervention was implemented in the Eastern Mediterranean. Most interventions were implemented in high-income countries (*n* = 27), and, of those, nearly all (*n* = 25) focused on MI, substance abuse, or both (Table 2). Interventions were evaluated using qualitative, quantitative, and mixed-methods (Table 1**)**.

**Table 2:** Summary of intervention characteristics (*N* = 42)

	HIV	MI	Substance abuse	MI + substance abuse	Total
WHO region					
Americas	2	12	1	1	16
European	–	5	1	–	6
Southeast Asian	2	1	–	–	3
African	2	1	–	–	3
Eastern Mediterranean	–	1	–	–	1
Western Pacific	3	9	–	1	13
Wealth of country*					
Lower middle income	4	3	–	–	7
Upper middle income	3	5	–	–	8
High income	2	21	2	2	27
Evaluation method					
Quantitative					
RCT	1	5	–	1	7
QE/C	–	6			6
QE/NC	3	14	1	1	19
Post survey, with control	–	1	–	–	1
RCX	1	–	–	–	1
Mixed methods					
RCT and qualitative	–	1	1	–	2
QE/C and process	1		–	–	1
QE/NC and qualitative	1	2	–	–	3
Process and qualitative	2		–	–	2

*Abbreviations*: *MI* mental illness, *RXS* repeated cross-sectional surveys, *QE/C* quasi-experimental with a control group, *QE/NC* quasi-experimental with no control group, *RCT* randomized controlled trial

*World Bank categorization

### Stigma reduction approaches utilized in identified interventions

Several key strategies to reduce stigma in healthcare settings emerged from the reviewed interventions.

1. “Provision of information” consisted of teaching participants about the condition itself or about stigma, its manifestations, and its effect on health.
2. “Skills-building activities” involved creating opportunities for healthcare providers to develop the appropriate skills to work directly with the stigmatized group.
3. “Participatory learning” approaches required participants (health facility staff or clients or both) to actively engage in the intervention.
4. “Contact with stigmatized group” relied on involving members of the stigmatized group in the delivery of the interventions to develop empathy, humanize the stigmatized individual, and break down stereotypes.
5. An “empowerment” approach was used to improve client coping mechanisms to overcome stigma at the health facility level.
6. “Structural” or “policy change” approaches included changing policies, providing clinical materials, redress systems, and facility restructuring.

Nearly every intervention took multiple approaches to reduce stigma (*n* = 24), except for two purely structural integration interventions [59, 60]. The most frequently used approach was contact with the stigmatized group (*n* = 30), but this was closely followed by provision of information (*n* = 29) and participatory learning (*n* = 28). Limited discernable patterns emerged across geographical regions, between lower middle-income countries and higher income countries, or in how interventions combined approaches. However, contact approaches were used in combination with most of the participatory learning interventions (21 of 28) and skills-building approaches (16 of 22) (Table 3).

**Table 3:** Approach by disease

Approach	HIV (9)	MI (29)	Substance abuse (2)	MI + substance abuse (2)	Total (42)
Information-based	7	20	1	1	29
Skills-building	4	16	1	1	22
Participatory learning	7	20	1	1	29
Contact	4	23	2	1	30
Empowerment	1	0	0	0	1
Structural	3	0	1	0	4

Note: some studies used multiple approaches

### How these approaches are delivered (methods)

Different ways of implementing the various approaches described above were used. Examples of the methods that can be used by each approach can be found in Table 4. Most interventions drew on multiple approaches and, consequently, also used multiple methods to deliver those approaches. Of the non-structural interventions, they were delivered in person, using video or streaming technology, or consisted of clinical placements, rotations, or clerkships for students. Such interventions were led or delivered by professionals (e.g., professors, expert medical providers, external facilitators) or clients (i.e., members of the stigmatized group). One was led by health facility staff members who had been trained as opinion leaders to champion stigma reduction [60–63]. Information provision approaches were delivered through didactic lectures, medical training courses, discussion, or printed educational materials. Contact approaches involved exposing the health facility staff participants to individuals living with the stigmatized condition, either in person or through videos, in non-clinical interactions. The mechanisms of these controlled exposures were through performances, discussions, participatory activities, or facilitated clinical placements. Participatory learning activities included discussion-based educational programs, interactive group work, role-playing, games, and assignments. Skills-building approaches were often operationalized through role-playing or through guided or controlled clinical practice, both with and without members of the stigmatized group.

**Table 4:** Intervention methods by approaches

	Information	Contact	Skills-building	Participatory learning	Structural	Empowerment
Educational materials	X					
Didactic lecture	X	X				
Performance	X	X				
Testimonials	X	X				
Discussion	X	X		X		
Interactive learning activities	X	X	X	X		
Clinic rotation	X	X	X	X		
Policies					X	
Protection materials or systems					X	
Task-shifting					X	
Service integration					X	
Counseling						X

We were unable to identify any discernable patterns of how methods or approaches were combined. Often, more passive activities, such as attending lectures or watching performances, were accompanied by open discussion or participatory activities. Of the four interventions that used structural approaches, three employed task-shifting—the redistribution of healthcare responsibilities to other sectors—and service integration. In two of these cases, HIV care was integrated into primary care, allowing HIV clients to integrate into the general patient pool and reduce their risk of status disclosure [59, 60]. Another structural intervention focused on reducing fear of HIV transmission as a means to reduce HIV-related stigma. This intervention trained facility-based stigma reduction popular opinion leaders on universal precaution procedures and provided infection protection supplies, such as gloves, to the whole facility [60–64]. Of the MI and substance abuse interventions that used clinical placements or role-playing to provide clinical care, six focused on recovery-oriented care. Recovery-oriented care is loosely characterized by a more optimistic view of recovery, empowerment of the patient, and aligning the providers’ goals with the clients’ recovery goals [65–70].

Four studies compared the effectiveness of different methods or approaches. Clarke et al. compared “dialectical behavioral therapy,” which aims to reduce prejudice and discrimination towards patients with personality disorders by providing staff with knowledge and skills to improve the effectiveness of their clinical practice, to “acceptance and commitment training,” which aims to provide self-management skills to reduce the impact of negative evaluations and strengthen value-driven behavior. For both types of training, staff attitudes improved and social distancing reduced, but they did not significantly differ [71]. Fernandez et al. compared the efficacy of an in-person, face-to-face contact plus educational lecture, to a video-based contact plus educational lecture. No significant differences were found between the two methods in terms of mental disorder stigma reduction [66]. Mak et al. compared the efficacy of an educational lecture plus a 90-min in-person sharing session led by people living with HIV to an educational lecture plus in-person interactive games led by research assistants (who were not living with HIV) and found no significant differences in HIV stigma reduction [72]. Winkler et al. compared an informational leaflet, a short video intervention, and a seminar involving direct contact with a mental health client. They found that attitudes and behavioral intent towards clients living with a mental disorder improved significantly for the video and seminar groups, whereas limited changes were seen for the flyer group. However, there were no significant differences between the groups [73].

### Stigma drivers targeted in interventions

Few articles explicitly identified the drivers targeted by their interventions. Li et al. targeted fear and fear-driven stigmatizing behaviors [60–63]. Batey et al. targeted attitudes, stigma knowledge, and HIV knowledge [74]. Geibel et al. targeted health facility policies and work environment and attitudes towards sexually active young people and people living with HIV [75]. Shah et al. targeted fear of, and misconceptions about, HIV transmission and attitudes towards populations vulnerable to HIV infection [76].

Interventions targeted attitudes, knowledge of stigma, knowledge of the condition, fear, ability to clinically manage the condition, client coping mechanisms, or institutional policies (Table 1). While some interventions explicitly stated the stigma driver targeted by their intervention, others did not; in cases where the stigma drivers were not explicitly described, we inferred the drivers targeted from the overall description of the intervention. Nearly 30 interventions targeted more than one driver. The most commonly targeted driver was knowledge about the condition. No regional trends or patterns were identified.

### Intervention efficacy

Of the 40 unique quantitative studies, 27 reduced stigma and 13 had mixed results (Table 1). However, the included interventions were evaluated using different measures, making cross-intervention comparisons difficult. Of note, certain interventions were evaluated using a wide array of stigma measures, while others were evaluated using just a few survey questions. Some evaluations had multiple follow-up surveys, while others only used one post-intervention time-point. Others pooled their measures of stigma into an overall index or score, while others examined differences between individual items. Interventions using more stigma measures were more likely to obtain mixed results than those using just a few measures.

## Discussion

### Gaps and opportunities for future research

Several gaps emerged from the literature search. Of the 42 unique studies, most (33) focused on MI or substance abuse and nine focused on HIV. Of note was the absence of recent stigma reduction interventions in health facilities for TB, diabetes, leprosy, or cancer. This may be because the presence of health facility stigma around diabetes and cancer has only relatively recently been recognized. For leprosy, it has very low and geographically confined prevalence. Possibly, interventions are being carried out, but are not being evaluated, or results have not yet been published or were published more than 5 years ago. The dearth of evaluations of stigma reduction interventions for TB was particularly notable; the lack of interventions addressing TB stigma has been noted by two other recent reviews of TB-related stigma [77, 78].

Other gaps identified included either no or few interventions that (1) targeted all levels of clinical or non-clinical health facility staff, concentrated on multiple ecological levels, or worked to structurally change physical or policy aspects of the facility environment; (2) engaged health facility staff and clients in a collaborative effort to design and implement stigma reduction interventions; (3) leveraged technology for interactive learning beyond videos for testimonials; and (4) recognized and addressed stigma experienced by health workers.

### Addressing health facility stigma at multiple levels

There is growing recognition that, to deliver a sustainable and scaled response to health facility stigma, it is important to address stigma at multiple ecological levels within a health facility [3, 64, 79]. While this search of the literature identified only one intervention targeting all levels of staff in a facility [64], current efforts led by some authors of this manuscript in Thailand (the 3X4 approach) [80], Ghana, and Tanzania (the Health Policy Project total facility approach) [81] are developing and testing a package of interventions that work at both the individual (health facility staff) and structural (health facility policy and environment) levels within a facility. At the individual level, these interventions focus on participatory training of health facility staff of all cadres (clinical and non-clinical). Any health facility employee who has client contact can stigmatize; therefore, working with all cadres of health workers is important. At the structural level, the 3X4 and the Health Policy Project total facility approaches are focused on developing and enforcing anti-discrimination policies, infection control by providing supplies and enforcing standard precaution infection control practices, as well as client complaint and compliment mechanisms. Further investigation of the potential for structural interventions to reduce stigma is needed [82], particularly around how the physical layout or space within a facility can contribute to, or mitigate, the experience and anticipation of stigma in facilities [83]. Based on the experiences of staff and clients, simple physical changes can lower the experience and risk of stigma, as well as unwanted disclosure [84, 85]. For example, a pharmacist participating in the stigma reduction training in Ghana became aware that their pharmacy inadvertently stigmatized clients living with HIV (and disclosed their HIV status) by having two separate windows for medicine pick-up: one for clients living with HIV, and one for everyone else. Following the intervention, all clients now go to the same window [86, 87].

### Bringing health workers and clients together for stigma reduction

Keeping those who fear, or are burdened, by stigmatization at the center of any response to stigma has been identified as a best practice [74, 84, 85, 88]. This includes working to empower people or groups experiencing stigma, for example, by building skills and efficacy to address internalized stigma and cope with and challenge stigma, and building partnerships with gatekeepers and opinion leaders for change. From the literature identified, the most common way of involving clients experiencing stigma in the intervention was as trainers or speakers [58, 64, 67–70, 72, 73, 76, 89–93]. The literature search only identified one intervention that went beyond this level of engagement to focus on an “empowerment” aspect [74]. This ongoing work in Alabama, USA, brings together health workers and clients in a workshop setting outside of the facility, to share information, increase contact, and use empowerment strategies to challenge HIV-related and intersecting stigmas. The latter is done by implementing a stigma reduction project that was developed by clients and health workers. Similarly, an ongoing intervention to prevent stigma towards people with MI or substance abuse in Lima, Peru, and Toronto, Canada, brings together primary health providers and clients to reduce stigma through five steps, one of which involves providers and clients working together in creative workshops to produce art that is presented to others [94].

### Utilizing technology for stigma reduction

In recent years, healthcare systems have witnessed rapid advances in technology, including, but not limited to, the use of electronic medical records and use of the internet, tablets, and phones to provide care, collect data, and support clinical information and ongoing education. These advances, particularly the use of self-learning via tablets, the Internet, and phones offer potentially efficient methods to deliver stigma reduction to busy health facility staff [73, 95]. Technology can also offer clients a way to mitigate or avoid health facility stigma [96, 97]. An ongoing study in India has developed, and is testing, a stigma reduction intervention that targets nursing students and health facility ward staff through two self-learning sessions on tablets, and one in-person 1.5-h group session, co-led by a person living with HIV [98]. This intervention targets several key individual-level drivers of stigma, including awareness, fear, and attitudes. Another co-author is leading the ongoing Client Centered Care Coordination (C4) intervention, which uses mobile technology to empower and help clients mitigate and avoid stigma in New York state (USA), Toronto (Canada), and multiple sites in Ghana [99]. This intervention uses different phone apps to connect clients living with HIV from key population communities, to peer support and to nurses and other health personnel, and to report and receive feedback on health behaviors and illness symptoms. Using mobile apps act as an access point to health services and reduce the opportunities for being exposed to stigma in the physical space of the health facility, as well as potential unwanted disclosure of HIV status.

### Reducing stigma towards healthcare workers

Lastly, we found no interventions with a specific focus on health workers living with a stigmatized disease, and addressing any stigma they may experience from co-workers or through the facility structures. Research has shown that stigma affects healthcare workers, either because of their own health status or as a result of working with stigmatized individuals [100, 101]. The HaTSaH study, an ongoing study in Free State province, South Africa, is addressing this gap through a combination intervention approach that focuses on reducing HIV and TB stigma among health workers towards colleague health workers living with HIV and TB through clinical, structural, and sociobehavioral factors [102].

Across these sets of ongoing efforts, which address different health condition stigmas, several factors are being recognized as key to the interventions. Involvement of clients living with the stigmatized condition or behavior is critical, whether this is by creating safe spaces for contact (e.g., panel discussions), as trainers, or as participants in joint provider–client workshops. It is critical to build facility management buy-in and ownership, while also creating and empowering facility-based “champion” teams of health facility workers and clients who develop and lead tailored stigma reduction efforts in their facilities. Additionally, it is important to pay attention to physical space and how it can lead to stigma and or unwanted disclosure of status.

## Limitations

There are several limitations to our literature review. We limited the focus of the review to seven specific conditions. The timeframe and scope are necessarily limited. Meta-analysis was not possible because of variability in study designs and a lack of standardized measures. Systematic reviews and meta-analyses are available for some of the specific health condition stigmas included in this paper, and we drew on these to contextualize the current analysis. Some interventions evaluated stigma using a single measure or question, while others measured many different stigma constructs using a host of measurement tools. As only articles published in English were included, completeness cannot be guaranteed.

Additionally, while there were many core similarities in how stigma could be addressed at the health facility level, regardless of disease, the generalizability of these findings to other conditions may be limited because identified interventions only addressed stigma related to HIV, MI, and substance abuse disorders, with a preponderance of interventions for the latter two conditions. Despite these limitations, the findings from the review draw from 42 stigma reduction efforts around the globe aimed at mitigating health facility stigma.

## Conclusion

Despite the ever-growing scientific evidence base on the prevalence of stigma in health facilities, and its negative impact on individuals’ health, relatively few interventions exist to address this major impediment in healthcare. This article highlights approaches and methods that have been used to reduce health condition stigma in health settings over the past 5 years, many of which are similar across different health condition stigmas. Particularly in resource-constrained health facilities, interventions that find synergies for stigma reduction across conditions could potentially create economies of scale, offering cost and time savings. The current state of knowledge regarding stigma reduction interventions provides a solid foundation to further develop interventions that address the gaps identified in this manuscript and address multiple health condition stigmas simultaneously. Future investment in stigma reduction should prioritize conditions that have been overlooked in the recent literature (for example, TB), rigorous evaluation, underrepresented geographic locations, addressing stigma at multiple ecological levels within a health facility for a sustainable response, and standardizing measures to facilitate comparisons between intervention approaches and methods.

Stigma does not only affect those who are living with stigmatized health conditions. Its ramifications reverberate outward through communities and inwards through the health facility into the policies and procedures that guide care, and on to the staff who are charged with providing care. It matters because reducing stigma has the potential to improve the health workplace environment, the quality of care provided by staff, the clinical outcomes of individuals living with stigmatized health conditions, and the social risks taken when accessing healthcare for particular conditions.

### Recommendations and future priorities

Future investment in research and health facility stigma reduction interventions should:

- Prioritize rigorous evaluation
- Standardize stigma measures to facilitate comparisons between intervention approaches and methods
- Study the scale-up and routinization of stigma reduction in health facilities, with a focus on sustainable responses
- Capture cost data on the interventions and include cost-effectiveness analysis
- Develop and test stigma reduction interventions tailored to the local context and culture that:
  - ○ Tackle multiple stigmas at once, while remaining attentive to the needs of individuals with specific health conditions or characteristics
  - ○ Focus on empowerment as an approach for clients or health workers to cope with or challenge stigma, and demand rights to stigma-free health services
  - ○ Recognize and address stigma experienced by health workers, including internalized and secondary stigma
  - ○ Target all levels of health facility staff, both clinical and nonclinical
  - ○ Leverage technology for interactive learning beyond video testimonials
  - ○ Work at a structural level to change the physical or policy aspects of the facility environment
  - ○ Concentrate on simultaneously targeting multiple ecological levels, such as targeting both individual attitudes and practices as well as the health facility policies and environment

## Abbreviations

MI: Mental illness
TB: Tuberculosis

## References

[1] Link BG, Phelan JC. Conceptualizing stigma. Annu Rev Soc. 2001;27(1):363–85.

[2] United Nations Agency for International Development (UNAIDS) (2000). Protocol for identification of discrimination against people living with HIV.

[3] Nyblade L, Stangl A, Weiss E, Ashburn K (2009). Combating HIV stigma in health care settings: what works?. J Int AIDS Soc.

[4] Ross CA, Goldner EM (2009). Stigma, negative attitudes and discrimination towards mental illness within the nursing profession: a review of the literature. J Psychiatr Ment Health Nurs.

[5] Hamann HA, Ostroff JS, Marks EG, Gerber DE, Schiller JH, Lee SJC (2014). Stigma among patients with lung cancer: a patient-reported measurement model. Psychooncology.

[6] Dodor EA, Kelly S, Neal K (2009). Health professionals as stigmatisers of tuberculosis: insights from community members and patients with TB in an urban district in Ghana. Psychol Health Med..

[7] Govindasamy D, Meghij J, Negussi EK, Baggaley RC, Ford N, Kranzer K (2014). Interventions to improve or facilitate linkage to or retention in pre-ART (HIV) care and initiation of ART in low and middle income settings – a systematic review. J Int AIDS Soc.

[8] Katz IT, Ryu AE, Onuegbu AG, Psaros C, Weiser SD, Bangsberg DR (2013). Impact of HIV-related stigma on treatment adherence: systematic review and meta-synthesis. J Int AIDS Soc.

[9] Musheke M, Ntalasha H, Gari S, Mckenzie O, Bond V, Martin-Hilber A (2013). A systematic review of qualitative findings on factors enabling and deterring uptake of HIV testing in sub-Saharan Africa. BMC Public Health.

[10] Chidyaonga-Maseko F, Chirwa ML, Muula AS (2015). Underutilization of cervical cancer prevention services in low and middle income countries: a review of contributing factors. Pan Afr Med J.

[11] Dey S (2014). Preventing breast cancer in LMICs via screening and/or early detection: the real and the surreal. World J Clin Oncol.

[12] Ettridge K, Bowden J, Chambers S, Smith D, Murphy M, Evans S (2018). “Prostate cancer is far more hidden…”: Perceptions of stigma, social isolation and help-seeking among men with prostate cancer. Eur J Cancer Care.

[13] Ng C, Lai P, Lee Y, Azmi S, Teo C (2015). Barriers and facilitators to starting insulin in patients with type 2 diabetes: a systematic review. Int J Clin Pract.

[14] Adams OP, Carter AO (2011). Knowledge, attitudes, practices, and barriers reported by patients receiving diabetes and hypertension primary health care in Barbados: a focus group study. BMC Fam Pract.

[15] Hofstraat K, van Brakel WH (2016). Social stigma towards neglected tropical diseases: a systematic review. Int Health.

[16] Wainberg ML, Scorza P, Shultz JM, Helpman L, Mootz JJ, Johnson KA (2017). Challenges and opportunities in global mental health: a research-to-practice perspective. Curr Psychiatr Rep.

[17] Lan CW, Lin C, Thanh DC, Li L (2018). Drug-related stigma and access to care among people who inject drugs in Vietnam. Drug Alcohol Rev.

[18] Stringer KL, Baker EH (2018). Stigma as a barrier to substance abuse treatment among those with unmet need: an analysis of parenthood and marital status. J Fam Issues.

[19] Storla DG, Yimer S, Bjune GA (2008). A systematic review of delay in the diagnosis and treatment of tuberculosis. BMC Public Health.

[20] Van Boekel LC, Brouwers EP, Van Weeghel J, Garretsen HF (2013). Stigma among health professionals towards patients with substance use disorders and its consequences for healthcare delivery: systematic review. Drug Alcohol Depend.

[21] Hatzenbuehler ML, O’Cleirigh C, Mayer KH, Mimiaga MJ, Safren SA (2011). Prospective associations between HIV-related stigma, transmission risk behaviors, and adverse mental health outcomes in men who have sex with men. Ann Behav Med.

[22] Rueda S, Mitra S, Chen S, Gogolishvili D, Globerman J, Chambers L (2016). Examining the associations between HIV-related stigma and health outcomes in people living with HIV/AIDS: a series of meta-analyses. BMJ Open.

[23] Murray SR, Kutzer Y, Habgood E, Murchie P, Walter FM, Mazza D (2017). Reducing barriers to consulting a general practitioner in patients at increased risk of lung cancer: a qualitative evaluation of the CHEST Australia intervention. Fam Pract.

[24] Teixeira ME, Budd GM (2010). Obesity stigma: a newly recognized barrier to comprehensive and effective type 2 diabetes management. J Am Acad Nurs Pract.

[25] Blixen CE, Kanuch S, Perzynski AT, Thomas C, Dawson NV, Sajatovic M (2016). Barriers to self-management of serious mental illness and diabetes. Am J Health Behav.

[26] Knaak S, Mantler E, Szeto A (2017). Mental illness-related stigma in healthcare: barriers to access and care and evidence-based solutions. Healthcare management forum: 2017.

[27] Yang LH, Wong LY, Grivel MM, Hasin DS (2017). Stigma and substance use disorders: an international phenomenon. Curr Opin Psychiatr.

[28] Bonadonna LV, Saunders MJ, Zegarra R, Evans C, Alegria-Flores K, Guio H (2017). Why wait? The social determinants underlying tuberculosis diagnostic delay. PLoS One.

[29] Kebede B, Abate T, Mekonnen D (2013). HIV self-testing practices among health care workers: feasibility and options for accelerating HIV testing services in Ethiopia. Pan Afr Med J.

[30] Siegel J, Yassi A, Rau A, Buxton JA, Wouters E, Engelbrecht MC (2015). Workplace interventions to reduce HIV and TB stigma among health care workers – where do we go from here?. Global Public Health..

[31] Khan R, Yassi A, Engelbrecht MC, Nophale L, van Rensburg AJ, Spiegel J (2015). Barriers to HIV counselling and testing uptake by health workers in three public hospitals in Free State Province, South Africa. AIDS Care.

[32] Van Brakel WH (2006). Measuring health-related stigma—a literature review. Psychol Health Med..

[33] Ogden J, Nyblade L (2005). Common at its core: HIV-related stigma across contexts.

[34] Scambler G (2009). Health-related stigma. Sociol Health Illness.

[35] Chang S, Cataldo J (2014). A systematic review of global cultural variations in knowledge, attitudes and health responses to tuberculosis stigma. Int J Tuberc Lung Dis..

[36] Ekstrand ML, Bharat S, Ramakrishna J, Heylen E (2012). Blame, symbolic stigma and HIV misconceptions are associated with support for coercive measures in urban India. AIDS Behav.

[37] Whittle HJ, Palar K, Ranadive NA, Turan JM, Kushel M, Weiser SD (2017). “The land of the sick and the land of the healthy”: disability, bureaucracy, and stigma among people living with poverty and chronic illness in the United States. Soc Sci Med.

[38] Courtwright A, Turner AN (2010). Tuberculosis and stigmatization: pathways and interventions. Public Health Rep.

[39] Abdoli S, Doosti Irani M, Hardy LR, Funnell M (2018). A discussion paper on stigmatizing features of diabetes. Nurs Open.

[40] Van Brakel W (2014). Stigma in leprosy: concepts, causes and determinants. Leprosy Rev.

[41] Cataldo JK, Slaughter R, Jahan TM, Pongquan VL, Hwang WJ (2011). Measuring stigma in people with lung cancer: psychometric testing of the cataldo lung cancer stigma scale. Oncol Nurs Forum.

[42] Daftary A (2012). HIV and tuberculosis: the construction and management of double stigma. Soc Sci Med.

[43] Straetemans M, Bakker M, Mitchell E (2017). Correlates of observing and willingness to report stigma towards HIV clients by (TB) health workers in Africa. Int J Tuberc Lung Dis..

[44] Fominaya AW, Corrigan PW, Rüsch N (2016). The effects of pity on self-and other-perceptions of mental illness. Psychiatry Res.

[45] Knapp S, Marziliano A, Moyer A (2014). Identity threat and stigma in cancer patients. Health Psychol Open.

[46] Nyblade L, Jain A, Benkirane M, Li L, Lohiniva AL, McLean R (2013). A brief, standardized tool for measuring HIV-related stigma among health facility staff: results of field testing in China, Dominica, Egypt, Kenya, Puerto Rico and St. Christopher & Nevis. J Int AIDS Soc.

[47] Stevelink S, Van Brakel W, Augustine V (2011). Stigma and social participation in Southern India: differences and commonalities among persons affected by leprosy and persons living with HIV/AIDS. Psychol Health Med.

[48] Fife BL, Wright ER (2000). The dimensionality of stigma: a comparison of its impact on the self of persons with HIV/AIDS and cancer. J Health Soc Behav.

[49] Rose S, Paul C, Boyes A, Kelly B, Roach D (2017). Stigma-related experiences in non-communicable respiratory diseases: a systematic review. Chron Respir Dis.

[50] Bowleg L (2012). The problem with the phrase women and minorities: intersectionality—an important theoretical framework for public health. Ame J Public Health.

[51] Bond V, Nyblade L (2006). The importance of addressing the unfolding TB-HIV stigma in high HIV prevalence settings. J Commun Appl Soc Psychol.

[52] Lekas H-M, Siegel K, Leider J (2011). Felt and enacted stigma among HIV/HCV-coinfected adults: the impact of stigma layering. Qual Health Res.

[53] Rudolph AE, Davis WW, Quan VM, Ha TV, Minh N, Gregowski A (2012). Perceptions of community-and family-level injection drug user (IDU)-and HIV-related stigma, disclosure decisions and experiences with layered stigma among HIV-positive IDUs in Vietnam. AIDS Care.

[54] PRISMA. Transparent reporting of systematic reviewd and meta-analyses: PRISMA; 2015. http://www.prisma-statement.org/. Accessed 20 Sept 2017

[55] Stangl AL, Lloyd JK, Brady LM, Holland CE, Baral S (2013). A systematic review of interventions to reduce HIV-related stigma and discrimination from 2002 to 2013: how far have we come?. J Int AIDS Soc.

[56] Downs SH, Black N (1998). The feasibility of creating a checklist for the assessment of the methodological quality both of randomised and non-randomised studies of health care interventions. J Epidemiol Commun Health.

[57] Spencer L, Ritchie J, Lewis J, Dillon L, National Centre for Social Research. Quality in qualitative evaluation: a framework for assessing research evidence. London: Government Chief Social Researcher’s Office; 2003.

[58] Knaak S, Hawke L, Patten S (2013). That’s just crazy talk evaluation report.

[59] Uebel K, Guise A, Georgeu D, Colvin C, Lewin S (2013). Integrating HIV care into nurse-led primary health care services in South Africa: a synthesis of three linked qualitative studies. BMC Health Serv Res.

[60] Odeny TA, Penner J, Lewis-Kulzer J, Leslie HH, Shade SB, Adero W (2013). Integration of HIV care with primary health care services: effect on patient satisfaction and stigma in rural Kenya. AIDS Res Treat.

[61] Li L, Liang LJ, Lin C, Wu Z (2015). Addressing HIV stigma in protected medical settings. AIDS Care.

[62] Li L, Liang LJ, Wu Z, Lin C, Guan J (2014). Assessing outcomes of a stigma reduction intervention with venue-based analysis. Soc Psychiatry Psychiatr Epidemiol.

[63] Li L, Lin C, Guan J, Wu Z (2013). Implementing a stigma reduction intervention in healthcare settings. J Int AIDS Soc.

[64] Pulerwitz J, Oanh KT, Akinwolemiwa D, Ashburn K, Nyblade L (2015). Improving hospital-based quality of care by reducing HIV-related stigma: evaluation results from Vietnam. AIDS Behav.

[65] Feeney L, Jordan I, McCarron P (2013). Teaching recovery to medical students. Psychiatr Rehabil J.

[66] Fernandez A, Tan KA, Knaak S, Chew BH, Ghazali SS (2016). Effects of brief psychoeducational program on stigma in Malaysian pre-clinical medical students: a randomized controlled trial. Acad Psychiatry.

[67] Flanagan EH, Buck T, Gamble A, Hunter C, Sewell I, Davidson L (2016). “Recovery speaks”: a photovoice intervention to reduce stigma among primary care providers. Psychiatr Serv.

[68] Happell B, Byrne L, Platania-Phung C, Harris S, Bradshaw J, Davies J (2014). Lived-experience participation in nurse education: reducing stigma and enhancing popularity. Int J Ment Health Nurs.

[69] Knaak S, Szeto A, Fitch K, Modgill G, Patten S (2015). Stigma towards borderline personality disorder: effectiveness and generalizability of an anti-stigma program for healthcare providers using a pre-post randomized design. Borderline Personal Disord Emot Dysregul.

[70] Papish A, Kassam A, Modgill G, Vaz G, Zanussi L, Patten S (2013). Reducing the stigma of mental illness in undergraduate medical education: a randomized controlled trial. BMC Med Educ..

[71] Clarke S, Taylor G, Bolderston H, Lancaster J, Remington B (2015). Ameliorating patient stigma amongst staff working with personality disorder: randomized controlled trial of self-management versus skills training. Behav Cogn Psychother.

[72] Mak WW, Cheng SS, Law RW, Cheng WW, Chan F (2015). Reducing HIV-related stigma among health-care professionals: a game-based experiential approach. AIDS Care.

[73] Winkler P, Janouskova M, Kozeny J, Pasz J, Mlada K, Weissova A (2017). Short video interventions to reduce mental health stigma: a multi-centre randomised controlled trial in nursing high schools. Soc Psychiatry Psychiatr Epidemiol.

[74] Batey DS, Whitfield S, Mulla M, Stringer KL, Durojaiye M, McCormick L (2016). Adaptation and implementation of an intervention to reduce HIV-related stigma among healthcare workers in the United States: piloting of the FRESH workshop. AIDS Patient Care STDs.

[75] Geibel S, Hossain SM, Pulerwitz J, Sultana N, Hossain T, Roy S (2017). Stigma reduction training improves healthcare provider attitudes toward, and experiences of, young marginalized people in Bangladesh. J Adolesc Health.

[76] Shah SM, Heylen E, Srinivasan K, Perumpil S, Ekstrand ML (2014). Reducing HIV stigma among nursing students: a brief intervention. West J Nurs Res.

[77] Sommerland N, Wouters E, Mitchell E, Ngicho M, Redwood L, Masquillier C (2017). Evidence-based interventions to reduce tuberculosis stigma: a systematic review. Int J Tuberc Lung Dis.

[78] Craig G, Daftary A, Engel N, O’Driscoll S, Ioannaki A (2017). Tuberculosis stigma as a social determinant of health: a systematic mapping review of research in low incidence countries. Int J Infect Dis.

[79] Rao D, Elshafei A, Nguyen M, Hatzenbueler ML, Frey S, Go V. Multi-level stigma interventions: state of the science and future directions. BMC Medicine. 10.1186/s12916-018-1244-y 10.1186/s12916-018-1244-y PMC6377735 30770756

[80] Srithanaviboonchai K, Stockton M, Pudpong N, Chariyalertsak S, Prakongsai P, Chariyalertsak C (2017). Building the evidence base for stigma and discrimination-reduction programming in Thailand: development of tools to measure healthcare stigma and discrimination. BMC Public Health.

[81] Health Policy Project (2011). Comprehensive package for reducing stigma and discrimination in health facilities.

[82] Corrigan PW, Schomerus G, Shuman V, Kraus D, Perlick D, Harnish A (2017). Developing a research agenda for reducing the stigma of addictions, part II: lessons from the mental health stigma literature. Am J Addict.

[83] Sullivan N (2012). Enacting spaces of inequality: placing global/state governance within a Tanzanian hospital. Space Cult.

[84] Novotná G, Urbanoski KA, Rush BR (2011). Client-centered design of residential addiction and mental health care facilities: staff perceptions of their work environment. Qual Health Res.

[85] Bil JS (2016). Stigma and architecture of mental health facilities. Br J Psychiatry.

[86] Topp SM, Chipukuma JM, Chiko MM, Matongo E, Bolton-Moore C, Reid SE (2012). Integrating HIV treatment with primary care outpatient services: opportunities and challenges from a scaled-up model in Zambia. Health Policy Plan.

[87] Health Policy Plus (2018). Webinar: How to engage with health facilities to reduce HIV-related stigma and move closer to test and treat goals.

[88] Heijnders M, Van Der Meij S (2006). The fight against stigma: an overview of stigma reduction strategies and interventions. Psychol Health Med..

[89] Hawke LD, Michalak EE, Maxwell V, Parikh SV (2014). Reducing stigma toward people with bipolar disorder: impact of a filmed theatrical intervention based on a personal narrative. Int J Soc Psychiatry.

[90] Jarvie AL, Buxton JA, Szeto AC, Austin JC. A pilot study of the effect of exposure to stand-up comedy performed by people with mental illness on medical students’ stigmatization of affected individuals. UBC Medical J. 2013;5(1)15–18.

[91] Jaworsky D, Gardner S, Thorne JG, Sharma M, McNaughton N, Paddock S (2017). The role of people living with HIV as patient instructors - reducing stigma and improving interest around HIV care among medical students. AIDS Care.

[92] Li J, Li J, Huang Y, Thornicroft G (2014). Mental health training program for community mental health staff in Guangzhou, China: effects on knowledge of mental illness and stigma. Int J Ment Health Syst.

[93] Lohiniva AL, Benkirane M, Numair T, Mahdy A, Saleh H, Zahran A, et al. HIV stigma intervention in a low-HIV prevalence setting: a pilot study in an Egyptian healthcare facility. AIDS Care. 2016;28(5):644–52. 10.1080/09540121.2015.1124974 26717980

[94] Khenti A, Mann R, Sapag JC, Bobbili SJ, Lentinello EK, van der Maas M (2017). Protocol: a cluster randomised control trial study exploring stigmatisation and recovery-based perspectives regarding mental illness and substance use problems among primary healthcare providers across Toronto. Ontario BMJ Open.

[95] Radhakrishna K, Dass D, Raj T, Rakesh D, Kishore R, Srinivasan K, et al. Development of a novel tablet-based approach to reduce HIV stigma among healthcare staff in India. Perspect Health Inform Manage. 2017;14(Spring):1b. PMC5430130 28566985

[96] Naslund J, Aschbrenner K, Marsch L, Bartels S (2016). The future of mental health care: peer-to-peer support and social media. Epidemiol Psychiatr Sci.

[97] Canidate S, Hart M (2017). The use of avatar counseling for HIV/AIDS health education: the examination of self-identity in avatar preferences. J Med Internet Res.

[98] Nyblade L, Srinivasan K, Mazur A, Raj T, Patil DS, Devadass D, et al. HIV stigma reduction for health facility staff: development of a blended-learning intervention. Front Public Health. 2018;6:165. 10.3389/fpubh.2018.00165 PMC6021510 29977887

[99] Nelson LE, Aaful G, Adu-Sarkodie Y, Agyarko-Poku T, Alio A, Boakye F (2018). Nurse-led mobile app-based symptom monitoring for HIV positive MSM in Ghana: client centered community care coordination (C5).

[100] Ha PN, Chuc NTK, Hien HT, Larsson M, Pharris A (2013). HIV-related stigma: impact on healthcare workers in Vietnam. Global Public Health.

[101] Lohiniva A-L, Kamal W, Benkirane M, Numair T, Abdelrahman M, Saleh H (2016). HIV stigma toward people living with HIV and health providers associated with their care: qualitative interviews with community members in Egypt. J Assoc Nurs AIDS Care.

[102] Rau A, Wouters E, Engelbrecht M, Masquillier C, Uebel K, Kigozi G (2018). Towards a health-enabling working environment-developing and testing interventions to decrease HIV and TB stigma among healthcare workers in the Free State, South Africa: study protocol for a randomised controlled trial. Trials.

[103] Aggarwal AK, Thompson M, Falik R, Shaw A, O’Sullivan P, Lowenstein DH (2013). Mental illness among us: a new curriculum to reduce mental illness stigma among medical students. Acad Psychiatry.

[104] Bamgbade BA, Barner JC, Ford KH (2017). Evaluating the impact of an anti-stigma intervention on pharmacy students’ willingness to counsel people living with mental illness. Commun Ment Health J.

[105] Bamgbade BA, Ford KH, Barner JC (2016). Impact of a mental illness stigma awareness intervention on pharmacy student attitudes and knowledge. Am J Pharmaceut Educ.

[106] Beaulieu T, Patten S, Knaak S, Weinerman R, Campbell H, Lauria-Horner B (2017). Impact of skill-based approaches in reducing stigma in primary care physicians: results from a double-blind, parallel-cluster, randomized controlled trial. Can J Psychiatr.

[107] Bingham H, O’Brien AJ (2018). Educational intervention to decrease stigmatizing attitudes of undergraduate nurses towards people with mental illness. Int J Ment Health Nurs.

[108] Economou M, Kontoangelos K, Peppou LE, Arvaniti A, Samakouri M, Douzenis A (2017). Medical students’ attitudes to mental illnesses and to psychiatry before and after the psychiatric clerkship: training in a specialty and a general hospital. Psychiatry Res.

[109] Friedrich B, Evans-Lacko S, London J, Rhydderch D, Henderson C, Thornicroft G (2013). Anti-stigma training for medical students: the education not discrimination project. Br J Psychiatry.

[110] Gulati P, Das S, Chavan BS (2014). Impact of psychiatry training on attitude of medical students toward mental illness and psychiatry. Indian J Psychiatry.

[111] Iheanacho T, Marienfeld C, Stefanovics E, Rosenheck RA (2014). Attitudes toward mental illness and changes associated with a brief educational intervention for medical and nursing students in Nigeria. Acad Psychiatry.

[112] Itzhaki M, Meridan O, Sagiv-Schifter T, Barnoy S (2017). Nursing students’ attitudes and intention to work with mentally ill patients before and after a planned intervention. Acad Psychiatry.

[113] Lyons Z, Janca A (2015). Impact of a psychiatry clerkship on stigma, attitudes towards psychiatry, and psychiatry as a career choice. BMC Med Educ.

[114] MacCarthy D, Weinerman R, Kallstrom L, Kadlec H, Hollander MJ, Patten S (2013). Mental health practice and attitudes of family physicians can be changed!. Perm J.

[115] Marzán-Rodríguez M, Varas-Díaz N, Neilands T (2015). Qualitative contributions to a randomized controlled trial addressing HIV/AIDS-stigma in medical students. Qual Rep.

[116] Michaels PJ, Corrigan PW, Buchholz B, Brown J, Arthur T, Netter C (2014). Changing stigma through a consumer-based stigma reduction program. Commun Ment Health J..

[117] Morawska A, Fletcher R, Pope S, Heathwood E, Anderson E, McAuliffe C (2013). Evaluation of mental health first aid training in a diverse community setting. Int J Ment Health Nurs.

[118] Moxham L, Taylor E, Patterson C, Perlman D, Brighton R, Sumskis S (2016). Can a clinical placement influence stigma? An analysis of measures of social distance. Nurs Educ Today.

[119] Muzyk AJ, Lentz K, Green C, Fuller S, May DB, Roukema L (2017). Emphasizing Bloom’s affective domain to reduce pharmacy students’ stigmatizing attitudes. Am J Pharmaceut Educ..

[120] Shen Y, Dong H, Fan X, Zhang Z, Li L, Lv H (2014). What can the medical education do for eliminating stigma and discrimination associated with mental illness among future doctors? Effect of clerkship training on chinese students’ attitudes. Int J Psychiatry Med.

[121] Wakeman SE, Kanter GP, Donelan K (2017). Institutional substance use disorder intervention improves general internist preparedness, attitudes, and clinical practice. J Addict Med.

